# Accidental Hypothermia in a Swiss Alpine Trauma Centre—Not an Alpine Problem

**DOI:** 10.3390/ijerph191710735

**Published:** 2022-08-29

**Authors:** Katrin Habegger, Simon Brechbühler, Karin Vogt, Jasmin S. Lienert, Bianca M. Engelhardt, Martin Müller, Aristomenis K. Exadaktylos, Monika Brodmann Maeder

**Affiliations:** 1 Department of Emergency Medicine, Inselspital, Bern University Hospital, University of Bern, 3010 Bern, Switzerland; 2 Hôpital du Valais, Spitalzentrum Oberwallis, 3930 Visp, Switzerland; 3 Department of Emergency Medicine, Fribourg Hospital, 1752 Villars-sur-Glâne, Switzerland; 4 Swiss Army, Military Medical Service, Regional Military Medical Center of Thun, 3600 Thun, Switzerland; 5 EURAC Research, Institute of Mountain Emergency Medicine, 39100 Bolzano, Italy

**Keywords:** hypothermia, temperature, drowning, trauma, mortality, extracorporeal life support ECLS

## Abstract

Background: Research in accidental hypothermia focuses on trauma patients, patients exposed to cold environments or patients after drowning but rarely on hypothermia in combination with intoxications or on medical or neurological issues. The aim of this retrospective single-centre cohort study was to define the aetiologies, severity and relative incidences of accidental hypothermia, methods of measuring temperature and in-hospital mortality. Methods: The study included patients ≥18 years with a documented body temperature ≤35 °C who were admitted to the emergency department (ED) of the University Hospital in Bern between 2000 and 2019. Results: 439 cases were included, corresponding to 0.32 per 1000 ED visits. Median age was 55 years (IQR 39–70). A total of 167 patients (38.0%) were female. Furthermore, 63.3% of the patients suffered from mild, 24.8% from moderate and 11.9% from severe hypothermia. Exposure as a single cause for accidental hypothermia accounted for 12 cases. The majority were combinations of hypothermia with trauma (32.6%), medical conditions (34.2%), neurological conditions (5.2%), intoxications (20.3%) or drowning (12.0%). Overall mortality was 22.3% and depended on the underlying causes, severity of hypothermia, age and sex.

## 1. Introduction

Accidental hypothermia, defined as an involuntary drop in core temperature ≤35 °C, is a common problem in environments with low ambient temperatures [1,2]. It is usually assigned to victims from avalanches, although hypothermia plays a minor role in the mortality of avalanche victims [3,4] Therefore, many publications on the care of patients suffering from accidental hypothermia originate from countries and regions with mountains and low ambient temperatures [5,6,7,8]. The environmental circumstances encountered by preclinical rescue teams may already suggest accidental hypothermia as a possible diagnosis in these patients and might cause a selection bias by overstating these “classical” circumstances.

Hypothermia also plays a prominent role in trauma: emergency physicians and traumatologists know the combination of accidental hypothermia and multiple trauma as the “trauma triad of death” [9]. This expression refers to the combination of hypothermia, coagulation disorders and acidosis, which increases the mortality of severely injured patients [10,11,12]. A large analysis of the National Trauma Data Bank (NTDB), the most extensive existing database of trauma cases, showed that patients with admission temperatures less than 35 °C had significantly greater mortality (25.5% vs. 3.0%, *p* < 0.001) than non-hypothermic patients [13]. In a metanalysis by Rösli et al., accidental hypothermia at admission was associated with significantly higher mortality in trauma patients (OR 5.18 [95% CI 2.61–10.28]) [14].

In 2010, researchers in Amsterdam published an article on accidental hypothermia in the Netherlands. In a single-centre study on hypothermic patients, they demonstrated an incidence of 11 cases per year and a median relative incidence of 0.32 per 1000 emergency department (ED) visits. In contrast to publications from alpine countries, they saw more patients who suffered from near-fatal drowning, neurological diseases or multiple trauma [15].

In 2013, a master thesis studied the characteristics of all patients who were admitted to the ED of the University of Bern Medical Centre (Inselspital Bern) between 2000 and 2012 [16]. As the catchment area of the ED is alpine, the authors assumed that many patients would be treated with accidental hypothermia due to activities in the mountains. In fact, however, there were more patients from urban areas who had suffered either medical emergencies or intoxication. Some patients from urban areas had also suffered trauma, especially traffic accidents. Mortality depended on age, sex and cause, as well as the severity of hypothermia. The same study team later decided to extend the observation period to twenty years (up until 2019), to better understand the underlying causes leading to accidental hypothermia and their impact on the survival of these patients. The extension of the primary study was conducted according to the design of the primary study from 2013.

The present single-centre retrospective cohort study aimed to describe (i) the relative incidence of accidental hypothermia in a large Swiss trauma centre and (ii) the methods of measuring temperature. Moreover, we aimed (iii) to determine the aetiologies and severity of accidental hypothermia, as well as (iv) in-hospital mortality. Our hypothesis was that more patients suffered from accidental hypothermia due to medical and neurological problems and that the incidence of hypothermia caused by exposure alone or avalanches is overestimated in the literature.

## 2. Materials and Methods

### 2.1. Study Setting

The ED of the University Hospital of Bern is one of the largest level I trauma centres in Switzerland that provides extracorporeal life support (ECLS). ECLS methods include extracorporeal rewarming by extracorporeal membrane oxygenation (ECMO), classical extracorporeal circulation (ECC) and a hybrid ECLS called Mini-ECC or MiECC [17]. All three methods can be applied for the invasive rewarming of patients suffering from severe accidental hypothermia [2]. Since 2005, all severely hypothermic patients have been treated according to an internally developed algorithm: This “Bernese hypothermia algorithm” was the first published guideline for decision-making in challenging situations of hypothermic cardiac arrest [18]. It is internally reviewed and constantly updated by a multidisciplinary team.

### 2.2. Eligibility Criteria

We included all patients aged ≥18 years—with no upper age limit—who were admitted to the ED of the University Hospital of Bern, Switzerland, and diagnosed with accidental hypothermia of any origin, defined as a body temperature ≤35 °C. Exclusion criteria were: therapeutic hypothermia, patients formerly diagnosed with hypothermia but admitted for other reasons, no measurement of body temperature available in the medical record and local freezing injuries without accompanying hypothermia.

### 2.3. Data Collection

A screening search in the medical records of the ED consultations was performed in the three databases that were used by physicians during the study period (KGA Digital, ARTS, UPTIME products AG, Zurich; Qualicare Office, Medical Database Software, Qualidoc AG, Bern, Switzerland, and E-Care, ED 2.1.3.0, Turnhout, Belgium). Search terms were the German expressions for “hypothermia”, “cooling”, “avalanche” or “freezing”. Data were anonymised by the research department of the ED and by the support office for scientific research, before being sent to the study group. Members of the study group then reviewed all of the medical charts and the defined underlying causes leading to hypothermia, based on the medical records of these patients.

### 2.4. Data Analysis

The following parameters were analysed: age, sex, date of consultation, underlying causes leading to accidental hypothermia, severity of accidental hypothermia, methods of measuring temperature and in-hospital mortality. Multiple underlying causes of accidental hypothermia were allowed if a principle cause for hypothermia could not be identified in the medical record or if two interacting aetiologies were identified (e.g., a hypothermic patient with stroke and hypoglycaemia).

On the basis of the extracted data, we were able to determine the absolute number of patients who suffered from accidental hypothermia, the relative incidence in relation to total admissions to the ED and factors influencing mortality, such as age, body temperature or cause of hypothermia.

The stages of accidental hypothermia were defined as mild (core temperature between 35 and 32 °C), moderate (core temperature between 31.9 and 28 °C) or severe (core temperature below 28 °C) [1,2].

### 2.5. Statistical Analysis

The statistical analysis was performed with STATA^®^ 16.1 (StataCorp, The College Station, TX, USA). For descriptive analysis, the distribution of continuous variables is presented using the median accompanied by the interquartile range (IQR, i.e., 25th to 75th percentile). The frequencies of categorical variables are shown with percent, accompanied by the absolute number. In order to understand the fluctuation over the years of the relative incidences of the number of hypothermia cases per 1000 ED visits, incidences were presented with a 95% confidence interval (CI) for proportions (the absolute number of ED visits as a denominator).

### 2.6. Ethical Considerations

The study was conducted in accordance with the Declaration of Helsinki. The protocol was approved by the Bern Ethical Committee (KEK No: 2017-00947 with an amendment until the end of 2019).

## 3. Results

**Figure 1:**
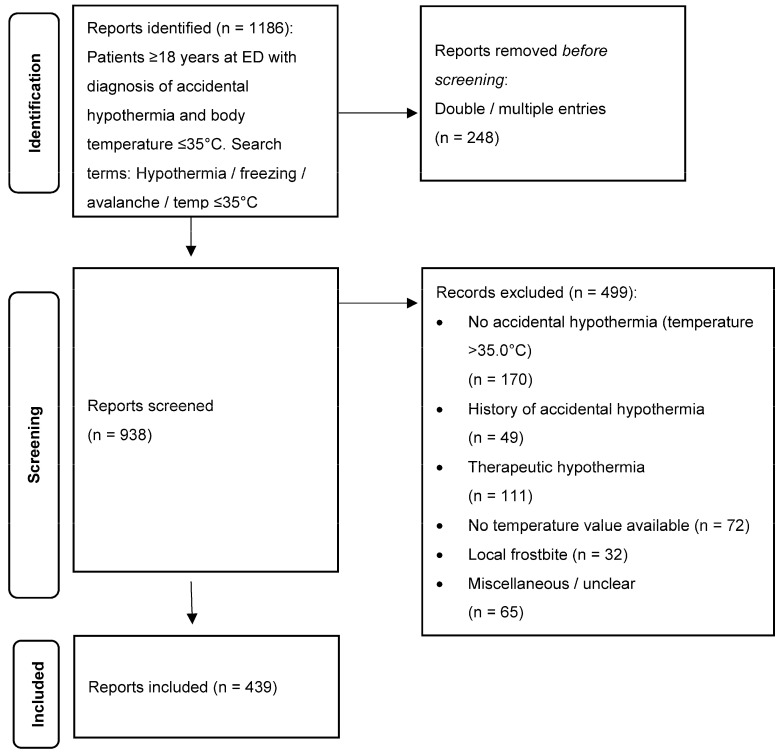
Flow diagram.

Within the search period from 2000 to 2019, we identified 1186 medical reports with the keyword “accidental hypothermia” or a body temperature of ≤35 °C. Two hundred and forty-eight (248) cases showed double or multiple entries, giving in all 938 cases for further analysis. Four hundred and ninety-nine (499) cases had to be excluded (for details see Figure 1). Sixty-five (65) cases labelled “miscellaneous or unclear” were mostly descriptions of local hypothermia, or hypothermia was said to be absent. Finally, 439 cases were then included in the study.

The median age of the whole cohort was 55 years (IQR 39–70). A total of 167 patients (38.0%) were female. Men had a median age of 53 years (IQR 37–66), and women had a median age of 62 years (IQR 46–77).

**Figure 2:**
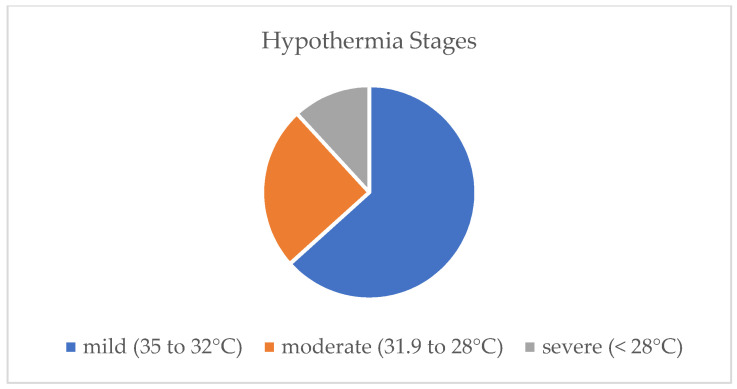
Hypothermia stages.

The annual incidence varied from one case in 2000 to a maximum of 48 cases in 2016, averaging 22 cases per year (Figure 2). The median yearly relative incidence (per 1000 ED visits) was 0.62 (95% CI: 0.47, 0.77). On average, over the years, every 1612nd (95% CI: 1.298, 2.127) ED visit was associated with accidental hypothermia. A total of 63.3% of our patients suffered from mild hypothermia (32.0–35.0 °C), 24.8% from moderate (28.0–31.9 °C) and 11.9% from severe hypothermia (<28.0 °C) (see Figure 2).

Aetiologies of accidental hypothermia are shown in Table 1. Primary hypothermia as a single cause of hypothermia (in the sense of exposure without any accompanying problem like trauma or medical illness) accounted for only 12 cases. Most of the patients suffered from secondary hypothermia, either in the context of trauma (32.6%) or medical conditions (34.2%). In 20.3% of all cases, intoxication was the leading cause for hypothermia, i.e., alcohol and drugs. Near-fatal drowning contributed to 12.0%, neurological cases to 5.2% and avalanche victims to 4.8% of the cases.

Age and sex distribution depended on the aetiology of hypothermia. Avalanche victims were predominantly male (85.7%) and had a median age of 38 years (IQR 33–48). The median age for intoxications and drowning accidents was 49 years (IQR 32–57); for trauma victims, it was 55 years (IQR 37–69); for patients with underlying medical issues, it was 65 years (IQR 50–77), and, for patients with underlying neurological conditions, it was 75 years (IQR 52–81). A total of 34.0% of drowning victims, 34.3% of trauma patients, 37.1% of intoxicated patients, 43.3% of patients with underlying medicinal problems and 60.9% of neurological patients were female. The median age was 55 years (IQR 45–69) for mild hypothermia and 57 years (IQR 43–69) for both moderate and severe hypothermia. A total of 109 patients (39.2%) in the group with mild hypothermia were female, as were 41 in the group with moderate (37.6%) and 17 in the group with severe accidental hypothermia (32.7%).

The measured body temperature of avalanche victims was significantly lower, with a median of 28.4 °C (IQR 26.0–31.4), compared to patients with intoxications or medical conditions, who had a median temperature of 32.2 °C (IQR 31.1–34.2). We identified five different methods for measuring temperature but only three for core temperature (oesophageal, rectal bladder); see Table 2. In 64.5% of our records, the method of measuring temperature was not mentioned. In the group of severely hypothermic cases, 53.8% of the measured temperatures were documented as core temperatures.

In-hospital mortality was 22.3% in our cohort and 19.8% for women and 23.9% for men. Mortality greatly depended on the underlying cause: intoxications showed a mortality of 5.6%. For near-fatal drowning, we found a mortality of 18.9%; for medical cases, we found a mortality of 24.0%; for trauma victims, we found a mortality of 26.6%; for neurological cases, we found a mortality of 39.1%, and, for avalanche victims, we found a mortality of 61.9%. In mild hypothermia, mortality was 16.2%; in moderate hypothermia, mortality was 30.3%, and, in severe hypothermia, mortality was 38.5%. In the age group >65 years, mortality increased to 25.3% compared to 10.7% in the group under 30 years and 23.2% in the group between 30 and 64 years.

## 4. Discussion

In this retrospective single-centre cohort study, we evaluated patients who suffered from accidental hypothermia and were admitted to the ED of the University Hospital of Bern between 2000 and 2019.

### 4.1. Demography

The age of the patients differed substantially between the subgroups: the avalanche victims were the youngest group, with a median age of 38, but patients with underlying medical or neurological problems were the oldest group, with median ages of 65 or 75 years, respectively. As the two last groups accounted for more than 40% of our cases, the median age of our cohort was unexpectedly high. The University Hospital of Bern has a large catchment area in alpine regions (Bernese Oberland and Valais). We had, therefore, expected to find younger patients suffering from accidental hypothermia caused during recreational activities in the cold mountains. We found only 21 avalanche victims, who were the youngest group but the group with the highest mortality. Only the already mentioned study from the Netherlands and two recent studies from Japan discussed the issue of elderly people who suffered from accidental hypothermia [15,19,20].

### 4.2. Relative Incidence

We found 439 cases of accidental hypothermia, defined as a body temperature ≤35 °C, corresponding to an incidence of 0.32 per 1000 emergency department (ED) visits. This relative incidence is in excellent accordance with the study by van der Ploeg [15].

### 4.3. Hypothermia Stages

Almost 2/3 of the cohort (63.3%) suffered from mild, 25% suffered from moderate and suffered 11.9% from severe accidental hypothermia. These data are in line with a study from Poland, wherein more than 75% of the patients suffered from mild, 16.4% from moderate and 8.2% from severe accidental hypothermia [8].

### 4.4. Aetiologies

Primary hypothermia, defined as exposure to low temperature as the sole reason for accidental hypothermia, was found in 12 cases only. However, in combination with other conditions including trauma, medical conditions or neurological events, exposure was mentioned in 23.7% of the 439 cases. This means that a vast majority of our patients suffered from secondary hypothermia with medical conditions causing or accompanying hypothermia. These findings are in line with our study hypothesis and the already mentioned articles from Japan and a recent publication from Denmark [21]. The same phenomenon is mentioned in two recent articles [22,23]. Most of our patients suffered from either trauma (32.6%) or medical conditions, most frequently hypoglycaemia (34.2%), which either led to or was combined with accidental hypothermia. Intoxications and near-fatal drowning or fatal drowning accidents were mentioned in 20.3%, or 12% of the cases, respectively. It was often not possible to define one leading aetiology in our cohort. Bearing in mind that the different causes are interdependent and might influence each other, we decided to register all possible factors. Their influence on the severity of the disease and their impact on mortality is difficult to define and needs further investigation.

### 4.5. Mortality

The overall in-hospital mortality in our cohort was high, 22%. It showed clear dependence on sex, age and the severity of hypothermia. Moreover, it clearly depended on the underlying cause. The question remains if and to what extent accidental hypothermia plays a role in the different groups. On the one hand, hypothermia can be (neuro-)protective in patients with hypoxemia or cardiac arrest [24] or deleterious in multiple trauma and especially in neurotrauma [11,14]. The first study based on data from the International Hypothermia Registry (IHR) showed a favourable outcome after hypothermic cardiac arrest, with an overall survival rate of 36% [7]. In contrast to our study, the study population of the IHR was much younger, and the authors discussed a possible selection bias when entering cases in the register. What this means for the group of hypothermic patients with a major medical condition remains unclear: they are older, with a median age of 62 years, and are mostly mildly to moderately hypothermic (median temperature 32.1 °C). We might therefore assume that either age or the underlying problem are primarily responsible for the poor outcome, rather than hypothermia. In our study, the most important factors for an unfavourable outcome were age, sex and the severity of hypothermia. The HOPE score, a score that was developed for severely hypothermic patients arriving in cardiac arrest, considers these factors as well and calculates a survival probability based on age, gender, core body temperature at onset, presence of arrhythmia at onset, cooling mechanism and the duration of cardiopulmonary resuscitation [25,26]. As the HOPE score was not used in the ED in Bern, we could not refer to these data, but it could be a very powerful instrument for further studies. These factors could also help to sort out multifactorial situations, wherein several factors may interact. A retrospective cohort study from Japan on urban hypothermia showed that—amongst others—age over 75 was associated with in-hospital mortality [27].

### 4.6. Temperature Measurement

A major issue was the documentation of the temperature: in more than 60% of the cases, no information about the method or the site of the measurement could be found in the medical records. An exception was the group of patients suffering from severe accidental hypothermia: in more than 50% of the cases, core temperature was documented.

It is understandable that core temperature is not always measured in mild hypothermia, but in more severe stages of accidental hypothermia, it may influence cardio-respiratory and neurological systems, so that the correct measurement of core temperature and its documentation should be the gold standard. ED personnel must be aware of the clinical picture of accidental hypothermia and must be sensitised to keeping in mind that most of our cohort were elderly people who primarily suffered from medical and neurological conditions.

### 4.7. Limitations

The study has several limitations caused by the setting as a single-centre study and the retrospective analysis, with limited information in the old records. We could not find a published standardised method for reporting the different aetiologies of accidental hypothermia and therefore had to decide upon an unvalidated list of clinical problems that could lead to accidental hypothermia. Another major problem was the incomplete documentation of the method of measuring temperature, as mentioned in the discussion. This issue has also been raised in the study by Van der Ploeg [15]. Therefore, we decided to use body temperature instead of core temperature.

## 5. Conclusions

Even though Switzerland, and specially the University Hospital in Bern, has a large catchment area in the alpine region, we found a cohort who were unexpectedly old and mainly suffered from medical or neurological conditions as an underlying cause of hypothermia.

Victims with primary hypothermia were rare. More than 3/4 of the cases were suffering from secondary hypothermia. This mostly occurred in combination with a medical condition, trauma or intoxication.

In 2/3 of the cases, the method or the site of the measured body temperature was not mentioned. Measuring core temperature, at least in severe hypothermia, should be the gold standard and should be correctly documented.

## Figures and Tables

**Table 1:** Aetiologies (multiple answers allowed).

Etiology	*n*	%
Exposure	104	23.7
Avalanche	21	4.8
Trauma	143	32.6
Drowning/Near—Drowning	53	12.1
Intoxication	89	20.3
Neurological event	23	5.2
Medical condition	150	34.2
Hypo-/Hyperglycaemia	36	8.2
Sepsis	13	3.0
Cardiac Arrest	27	6.2
Others	40	9.1
Unknown	34	7.7

**Table 2:** Methods of measuring temperature.

Measurement Method	*n*	%	Measurement Type
Oesophagal	25	5.7	Body core temperature
Rectal	33	7.5	
Bladder	10	2.3	
Body core temperature (not further specified)	26	5.9	
Ear	37	8.4	Peripheral body temperature
Axillary	16	3.6	
Other peripheral body temperature	9	2.1	
Unknown	283	64.5	Not defined

## Data Availability

Data supporting reported results are available from the authors.

## References

[1] Brown D.J., Brugger H., Boyd J., Paal P. (2012). Accidental hypothermia. N. Engl. J. Med..

[2] Paal P., Gordon L., Strapazzon G., Brodmann Maeder M., Putzer G., Walpoth B., Wanscher M., Brown D., Holzer M., Broessner G. (2016). Accidental hypothermia–an update. Scand. J. Trauma Resusc. Emerg. Med..

[3] Boyd J., Haegeli P., Abu-Laban R.B., Shuster M., Butt J.C. (2009). Patterns of death among avalanche fatalities: A 21-year review. CMAJ.

[4] Procter E., Strapazzon G., Dal Cappello T., Zweifel B., Würtele A., Renner A., Falk M., Brugger H. (2016). Burial duration, depth and air pocket explain avalanche survival patterns in Austria and Switzerland. Resuscitation.

[5] Brändström H., Johansson G., Giesbrecht G.G., Ängquist K.-A., Haney M.F. (2014). Accidental cold-related injury leading to hospitalization in northern Sweden: An eight-year retrospective analysis. Scand. J. Trauma Resusc. Emerg. Med..

[6] Pasquier M., Taffé P., Kottmann A., Mosimann U., Reisten O., Hugli O. (2014). Epidemiology and mortality of glacier crevasse accidents. Injury.

[7] Walpoth B.H., Maeder M.B., Courvoisier D.S., Meyer M., Cools E., Darocha T., Blancher M., Champly F., Mantovani L., Lovis C. (2021). Hypothermic Cardiac Arrest–Retrospective cohort study from the International Hypothermia Registry. Resuscitation.

[8] Kosiński S., Darocha T., Gałązkowski R., Drwiła R. (2015). Accidental hypothermia in Poland–estimation of prevalence, diagnostic methods and treatment. Scand. J. Trauma Resusc. Emerg. Med..

[9] Mikhail J. (1999). The trauma triad of death: Hypothermia, acidosis, and coagulopathy. AACN Adv. Crit. Care.

[10] Tsuei B.J., Kearney P.A. (2004). Hypothermia in the trauma patient. Injury.

[11] Mommsen P., Andruszkow H., Frömke C., Zeckey C., Wagner U., Van Griensven M., Frink M., Krettek C., Hildebrand F. (2013). Effects of accidental hypothermia on posttraumatic complications and outcome in multiple trauma patients. Injury.

[12] Weuster M., Brück A., Lippross S., Menzdorf L., Fitschen-Oestern S., Behrendt P., Iden T., Höcker J., Lefering R., Seekamp A. (2016). Epidemiology of accidental hypothermia in polytrauma patients: An analysis of 15,230 patients of the TraumaRegister DGU. J. Trauma Acute Care Surg..

[13] Martin R.S., Kilgo P.D., Miller P.R., Hoth J.J., Meredith J.W., Chang M.C. (2005). Injury-Associated Hypothermia: An Analysis of the 2004 National Trauma Data Bank. Shock.

[14] Rösli D., Schnüriger B., Candinas D., Haltmeier T. (2020). The impact of accidental hypothermia on mortality in trauma patients overall and patients with traumatic brain injury specifically: A systematic review and meta-analysis. World J. Surg..

[15] van der Ploeg G.-J., Goslings J.C., Walpoth B.H., Bierens J.J. (2010). Accidental hypothermia: Rewarming treatments, complications and outcomes from one university medical centre. Resuscitation.

[16] Habegger K. (2013). Akzidentelle Hypothermie an einem Zentrumsspital; Datenaufarbeitung vom 1 January 2000–31 March 2012. Master’s Thesis.

[17] Anastasiadis K., Antonitsis P., Haidich A.-B., Argiriadou H., Deliopoulos A., Papakonstantinou C. (2013). Use of minimal extracorporeal circulation improves outcome after heart surgery; a systematic review and meta-analysis of randomized controlled trials. Int. J. Cardiol..

[18] Maeder M.B., Dünser M., Eberle B., Loetscher S., Dietler R., Englberger L., Martinolli L., Neumann M., Stalder M., Exadaktylos A. (2011). The Bernese Hypothermia Algorithm: A consensus paper on in-hospital decision-making and treatment of patients in hypothermic cardiac arrest at an alpine level 1 trauma centre. Injury.

[19] Matsuyama T., Morita S., Ehara N., Miyamae N., Okada Y., Jo T., Sumida Y., Okada N., Watanabe M., Nozawa M. (2018). Characteristics and outcomes of accidental hypothermia in Japan: The J-Point registry. Emerg. Med. J..

[20] Morita S., Matsuyama T., Ehara N., Miyamae N., Okada Y., Jo T., Sumida Y., Okada N., Watanabe M., Nozawa M. (2018). Prevalence and outcomes of accidental hypothermia among elderly patients in Japan: Data from the J-Point registry. Geriatr. Gerontol. Int..

[21] Wiberg S., Mortensen A.F., Kjaergaard J., Hassager C., Wanscher M. (2021). Accidental hypothermia in Denmark: A nationwide cohort study of incidence and outcomes. BMJ Open.

[22] Paal P., Pasquier M., Darocha T., Lechner R., Kosinski S., Wallner B., Zafren K., Brugger H. (2022). Accidental hypothermia: 2021 update. Int. J. Environ. Res. Public Health.

[23] Paal P., Rauch S. (2018). Indoor accidental hypothermia in the elderly: An emerging lethal entity in the 21st century. Emerg. Med. J..

[24] Yenari M.A., Han H.S. (2012). Neuroprotective mechanisms of hypothermia in brain ischaemia. Nat. Rev. Neurosci..

[25] Pasquier M., Hugli O., Paal P., Darocha T., Blancher M., Husby P., Silfvast T., Carron P.-N., Rousson V. (2018). Hypothermia outcome prediction after extracorporeal life support for hypothermic cardiac arrest patients: The HOPE score. Resuscitation.

[26] Pasquier M., Rousson V., Darocha T., Bouzat P., Kosiński S., Sawamoto K., Champigneulle B., Wiberg S., Wanscher M.C.J., Maeder M.B. (2019). Hypothermia outcome prediction after extracorporeal life support for hypothermic cardiac arrest patients: An external validation of the HOPE score. Resuscitation.

[27] Okada Y., Matsuyama T., Morita S., Ehara N., Miyamae N., Jo T., Sumida Y., Okada N., Kitamura T., Iiduka R. (2019). Prognostic factors for patients with accidental hypothermia: A multi-institutional retrospective cohort study. Am. J. Emerg. Med..

